# Enhanced Real-Time Highway Object Detection for Construction Zone Safety Using YOLOv8s-MTAM

**DOI:** 10.3390/s25206420

**Published:** 2025-10-17

**Authors:** Wen-Piao Lin, Chun-Chieh Wang, En-Cheng Li, Chien-Hung Yeh

**Affiliations:** 1 Department of Electrical Engineering, Chang Gung University, Taoyuan 33303, Taiwan; m0921107@cgu.edu.tw (C.-C.W.); m1321017@mail.cgu.edu.tw (E.-C.L.); 2 Department of Electrical Engineering, Ming Chi University of Technology, New Taipei City 243303, Taiwan; 3 Department of Photonics, Feng Chia University, Taichung 40724, Taiwan; yeh1974@gmail.com

**Keywords:** YOLOv8, object detection, motion-temporal attention, autonomous driving, construction vehicle, warning sign, data augmentation

## Abstract

Reliable object detection is crucial for autonomous driving, particularly in highway construction zones where early hazard recognition ensures safety. This paper introduces an enhanced YOLOv8s-based detection system incorporating a motion-temporal attention module (MTAM) to improve robustness under high-speed and dynamic conditions. The proposed architecture integrates a cross-stage partial (CSP) backbone, feature pyramid network-path aggregation network (FPN-PAN) feature fusion, and advanced loss functions to achieve high accuracy and temporal consistency. MTAM leverages temporal convolutions and attention mechanisms to capture motion cues, enabling effective detection of blurred or partially occluded objects. A custom dataset of 34,240 images, expanded through extensive data augmentation and 9-Mosaic transformations, is used for training. Experimental results demonstrate strong performance with mAP(IoU[0.5]) of 90.77 ± 0.68% and mAP(IoU[0.5:0.95]) of 70.20 ± 0.33%. Real-world highway tests confirm recognition rates of 96% for construction vehicles, 92% for roadside warning signs, and 84% for flag bearers. The results validate the framework’s suitability for real-time deployment in intelligent transportation systems.

## 1. Introduction

In recent years, the rapid advancement of artificial intelligence (AI) has greatly accelerated progress in autonomous driving technologies [1,2]. The development of autonomous vehicle systems critically depends on their ability to detect, recognize, and classify objects, as these capabilities directly affect vehicle navigation and road safety [3]. With continuous improvements in computing hardware and deep learning algorithms, significant progress has been made in vision-based object detection for vehicles. Such advancements are essential in intelligent transportation systems (ITS), automated surveillance, and autonomous driving, where real-time performance and high detection accuracy are crucial.

Among the most widely adopted deep-learning-based frameworks for object detection are the single-shot multi-box detector (SSD) [4], RetinaNet [5], and the you only look once (YOLO) series [6,7]. Although these models share a common objective—accurate object detection—they differ substantially in network design and the trade-offs between detection speed and accuracy. The SSD architecture achieves real-time detection by leveraging multi-scale feature maps and predefined bounding boxes of various sizes, eliminating the need for a region proposal stage, which makes it efficient for embedded and mobile applications [8]. RetinaNet balances speed and accuracy by combining a backbone network with a feature pyramid network (FPN) for multi-scale feature extraction and introduces focal loss to address class imbalance [9]. The YOLO series, particularly YOLOv5 and YOLOv8, represents the latest advances in single-stage detection. YOLOv5 employs cross-stage partial (CSP) modules to improve feature reuse and computational efficiency, while YOLOv8 adopts anchor-free prediction, decoupled classification–regression heads, and enhanced data augmentation strategies, enabling real-time inference with accuracy comparable to or exceeding that of two-stage detectors [10].

These object detection models have been extensively applied in vehicle-related tasks such as traffic sign recognition, vehicle counting, pedestrian and cyclist detection, and hazard identification in construction zones. To improve robustness under real-world conditions, modern training pipelines now incorporate advanced data augmentation (e.g., Mosaic, Mixing, and random affine transformations), refined loss functions (e.g., CIoU, EIoU), and multi-scale feature fusion networks such as FPN and PANet [11]. With the emergence of powerful hardware accelerators such as the NVIDIA RTX 40-series GPUs and Apple M1/M2 chips, and the increasing adoption of lightweight models like YOLONAS and MobileNetV3-based SSD, real-time object detection is now feasible on embedded and edge devices. Current research continues to focus on improving detection accuracy, reducing inference latency, enabling domain adaptation, and developing few-shot learning approaches for previously unseen object categories [12,13].

According to Taiwanese highway safety regulations [14], all construction vehicles performing maintenance work on expressways must be equipped with construction warning signs and electronic flag bearers [15,16]. These safety devices are designed to alert approaching drivers and reduce the risk of collisions within active construction zones. However, existing YOLO-based detection systems still face major challenges in real-world, dynamic highway environments, including motion blur, occlusion, illumination changes, rapid movement, and temporal inconsistency, all of which degrade the detection accuracy of single-frame models such as YOLOv8s. Furthermore, construction-zone scenarios are under-represented in existing datasets, leading to limited model generalization capability. To address these issues, recent studies have proposed various temporal attention mechanisms (TAMs) [17] to enhance predictive performance across both spatial and temporal dimensions. Some works have adopted lightweight temporal convolution–CNN hybrid architectures [18], such as the LTA-CNN, for real-time edge-computing applications. In addition, other researchers have explored space–time mixed-attention transformers [19], spatiotemporal attention with 3D convolution [20], and video saliency prediction via single feature enhancement and temporal recurrence [21]. While these approaches improve temporal modeling, they often introduce high computational cost or require large-scale datasets for effective training.

To achieve a balance between detection accuracy, computational efficiency, and cost in high-speed highway object detection and recognition, this study proposes a motion–temporal attention module (MTAM) that integrates lightweight 3D temporal convolution, residual fusion, and channel–spatial attention, embedded within the YOLOv8s backbone. The MTAM enables motion-robust, low-latency detection suitable for real-world highway construction environments. The overall architecture combines a CSP backbone, FPN for multi-scale feature extraction, and CIoU/EIoU loss functions, maintaining high accuracy even under partial occlusion, dynamic lighting, and high-speed motion. The model is trained to detect three key object categories: construction vehicles and signs, roadside warning signs, and electronic flag bearers. These objects serve as critical safety indicators of roadwork activity, helping both human drivers and intelligent vehicles identify hazardous zones early and take appropriate actions. By enhancing environmental perception, the proposed system provides practical benefits for the development of intelligent transportation systems (ITS) and significantly improves the operational safety and reliability of autonomous driving technologies under challenging expressway conditions.

## 2. Neural Network Architecture

**Figure 1:**
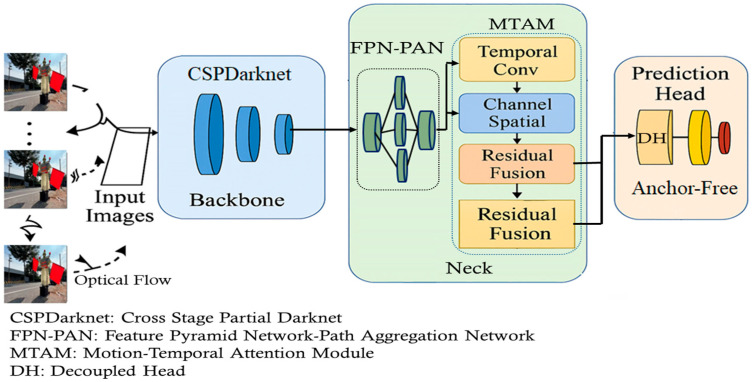
Modified YOLOv8 network architecture.

An improved YOLOv8s-based detection architecture, shown in Figure 1, is tailored for real-time and robust performance in highway and autonomous driving scenarios. The design preserves the core CSPDarknet backbone of YOLOv8s while introducing key enhancements in the Neck and Prediction Head to improve temporal consistency and detection accuracy under high-speed conditions. The backbone employs CSPDarknet, composed of convolutional and CSP layers, to extract low- and mid-level features with efficient gradient flow and reduced computational cost. These features are then passed to the Neck, where a combined FPN-PAN structure integrates multi-resolution representations. A central innovation here is the Motion-Temporal Attention Module (MTAM), which captures temporal cues across short frame sequences to improve detection of blurred or fast-moving objects [22,23].

Finally, the prediction head consists of a decoupled head (DH) and an anchor-free mechanism. The DH separates classification from bounding box regression, while the anchor-free design regresses object centers and boundaries directly using CIoU/EIoU losses [24]. This reduces complexity and improves generalization to diverse object shapes. Implemented in PyTorch, the lightweight model achieves up to 92 fps and is optimized with data augmentation strategies, making it suitable for embedded intelligent transportation systems.

### 2.1. Motion-Temporal Attention Module (MTAM)

MTAM is inserted in the Neck of the YOLOv8s architecture, parallel to or following the FPN-PAN module as shown in Figure 1. In the baseline YOLOv8s architecture, the Neck stage (FPN-PAN) aggregates multi-scale features from the backbone layers (P3, P4, P5). To enhance temporal modeling for video-based object detection, we integrate the Motion-Temporal Attention Module (MTAM) into the Neck stage. Specifically, MTAM is inserted between the P4 and P5 feature maps after feature aggregation but before these features are passed to the detection head [25,26].

The input to MTAM is a temporal sequence of seven consecutive frames, where each frame’s feature maps have the following dimensions: P4 = [Batch, 256, H/16, W/16] and P5 = [Batch, 512, H/32, W/32]. MTAM applies 3D temporal convolution with kernel size 3 × 3 × 3 and stride (1,1,1) along the temporal dimension, followed by residual fusion and channel–spatial attention mechanisms. The output of MTAM preserves the same spatial resolution as the input while embedding temporal dependencies. These enriched features are then reintroduced into the FPN-PAN for further top-down and bottom-up aggregation before being passed to the YOLOv8s detection head. By situating MTAM at this stage, the model effectively leverages temporal context to improve detection of motion-blurred or partially occluded objects while maintaining compatibility with the original pyramid flow.

It integrates both spatial attention and temporal attention to selectively emphasize informative regions over a sequence of frames. Let the input feature sequence be denoted by

(1) $$F=\left\{f_{1},f_{2},\ldots \ldots ,f_{T}\right\},\;f_{t}\in R^{C\times H\times W}$$

Temporal attention computes importance weights across time:

(2) $$\alpha_{t}=\frac{exp(\emptyset \left(f_{t}\right))}{\sum_{1}^{T}exp(\emptyset \left(f_{t}\right))}$$

where *ϕ* is temporal projection function. The output is a weighted sum over time:

(3) $$F_{temp}=\sum_{t=1}^{T}\alpha_{t}f_{t}$$

A spatial attention mask *A_s_* ∈ R*^H×W^* can be computed as

(4) $$A_{s}=\sigma ({Conv}_{1\times 1}\left(f_{t}\right))$$

Final attention-enhanced feature:

(5) $$F_{MTAM}=A_{s}⊙F_{temp}+F_{temp}$$

where *σ* is the sigmoid function, and ⊙ denotes element-wise multiplication.

**Figure 2:**
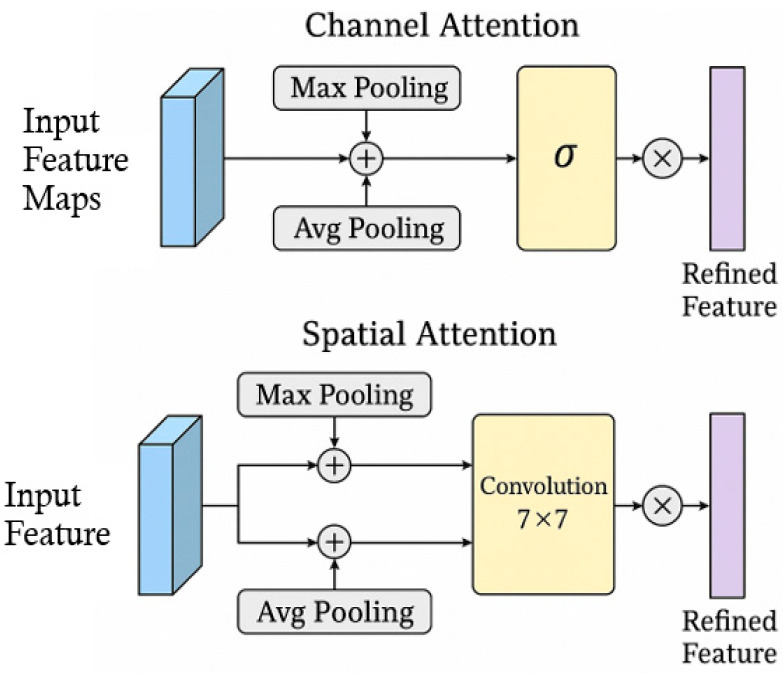
The structure of convolutional block attention module (CBAM).

The algorithm steps for the MTAM are designed to enhance object detection in high-speed driving environments by capturing temporal and motion cues from sequential frames. First, a sequence of consecutive input images is passed through the YOLOv8 backbone to extract deep feature maps for each frame. These feature maps are then stacked along the temporal dimension and processed using a temporal convolution layer, which captures motion patterns and dynamic changes across frames. Optionally, optical flow information can be incorporated to guide motion-aware feature alignment. Next, a combined channel and spatial attention mechanism—inspired by convolutional block attention module (CBAM) as shown in Figure 2—is applied to emphasize important features and suppress noise. Channel attention is computed by aggregating global information across spatial dimensions, while spatial attention focuses on the spatial regions that are most relevant for detection. The attention-weighted features are then fused with the original Neck outputs through residual connections, enabling the model to retain both motion-aware and spatially rich features. Moreover, residual fusion is implemented as a two-stage structure to progressively enhance feature integration. The first residual fusion combines dynamic features extracted from temporal convolution with static spatial features derived from the channel–spatial attention mechanism, ensuring effective collaboration of multi-source information. Subsequently, the second residual fusion further refines and strengthens the fused representation, preserving original feature information while mitigating gradient vanishing issues, thereby improving network stability. This final motion-temporal enhanced representation is forwarded to the anchor-free prediction head, enabling more robust detection of fast-moving or blurred objects such as construction vehicles and signs, flag bearers or roadside warning signs, which are common in real-world highway conditions.

This module can be integrated into the YOLOv8s detection pipeline before the detection head to enhance both temporal awareness and context-adaptive feature representation, thereby improving detection robustness in highway construction environments.

### 2.2. Loss Functions

Object detection accuracy is commonly evaluated using intersection over union (IoU), defined as the overlap-to-union ratio between predicted and ground-truth boxes. Extended metrics include generalized IoU (GIoU), complete IoU (CIoU), and efficient IoU (EIoU) [27,28]:

(6) $$GIoU=IoU-\frac{\left|C-\left(A\cup B\right)\right|}{\left|C\right|}$$

(7) $$CIoU=IoU-\frac{\rho^{2}\left(b,b^{*}\right)}{c^{2}}+\alpha v$$

(8) $$EIoU=IoU-\frac{\rho^{2}\left(b,b^{*}\right)}{c^{2}}+\frac{|w-w^{*}|}{w^{*}}+\frac{|h-h^{*}|}{h^{*}}$$

Here, *IoU* = |*A*∩*B*|/|*A*∪*B*|; |*C*| is the smallest enclosing box; *ρ* is the center distance; *c* is the diagonal length; v measures aspect ratio; and α is a trade-off weight. (*w*, *h*) and (*w*, h*) are widths and heights of predicted and ground-truth boxes. Performance is further assessed with precision and recall:

*Recall* = *TP*/(*TP* + *FN*), (9)

*Precision* = *TP*/(*TP* + *FP*) (10)

where *TP* are the true cases, *FP* are the false positive cases, and *FN* are the false negative cases. Mean average precision (mAP) provides a standard benchmark: mAP(IoU[0.5]) uses IoU = 0.5, while mAP(IoU[0.5:0.95]) averages over thresholds 0.5–0.95.

These advanced IoU variants overcome the limitations of basic IoU, addressing non-overlapping boxes, center distance, and aspect ratio. By integrating GIoU, CIoU, and EIoU, YOLOv8 achieves more robust optimization and higher detection accuracy under diverse conditions.

### 2.3. Improved YOLOv8s Loss Function with MTAM Module

We propose an enhanced total loss function defined as:

(11) $$L_{total}=\lambda_{box}L_{EIoU}+\lambda_{cls}L_{cls}+\lambda_{dfl}L_{dfl}+\lambda_{MTAM}L_{MTAM}$$

(12) $$L_{EIoU}=1-IoU-\frac{\rho^{2}\left(b,b^{*}\right)}{c^{2}}+\frac{|\omega -\omega^{*}|}{\omega^{*}}+\frac{|h-h^{*}|}{h^{*}}$$

where *L*_EIoU_ is the efficient IoU loss, *L*_cls_ is classification loss, *L*_dfl_ is distribution focal loss, *L*_MTAM_: temporal-attention-guided supervision, enforcing temporal consistency:

(13) $$L_{MTAM}=\sum_{t=1}^{T-1}{\left\|f_{t}^{att}-f_{t+1}^{att}\right\|}^{2}$$

where *f_t_* is the attention-weighted feature from MTAM at frame t.

## 3. Experimental Results

Establishing a robust experimental environment is essential for the efficient development and deployment of deep learning models. It ensures an optimal balance between powerful hardware and a compatible, up-to-date software stack, enabling high-performance experimentation, model training, and algorithm testing with enhanced stability and reliability.

### 3.1. Experimental Environment Setup

All training experiments in this study were conducted on a workstation equipped with a 6-core AMD Ryzen 5 7500F 64-bit processor (3.7 GHz, 32 GB RAM) and an NVIDIA GeForce RTX 5080 GPU featuring 32 GB of dedicated video memory. The system operated under Microsoft Windows 11 Home and utilized the PyTorch 2.1.2 deep learning framework with CUDA 12.4 and Python 3.13.2 for GPU-accelerated computation. Model training employed the stochastic gradient descent (SGD) optimizer (momentum = 0.937, weight decay = 5 × 10^−4^) with a cosine-annealing learning-rate schedule and a 5-epoch warm-up. The initial learning rate, batch size, and total epochs were set to 0.01, 24, and 150, respectively. The confidence threshold and NMS IoU were fixed at 0.25 and 0.45. Detailed specifications of the experimental environment are summarized in Table 1.

The real-time image inference framework in this study adopts the NVIDIA Jetson Nano super embedded platform, which supports the TensorRT deep learning inference acceleration engine. This research is based on PyTorch and integrates a customized deep learning model combining YOLOv8, MTAM, and OpenCV. First, MTAM is embedded into the YOLOv8 architecture. This module is implemented in PyTorch using 3D convolution, channel-spatial attention mechanisms, and residual fusion layers. MTAM is inserted between the YOLOv8 backbone and detection head, and the forward function is modified to accept time-series image input with a shape of [B, C, T, H, W]. Next, OpenCV is used to capture real-time images from the dashcam, and each frame is resized, normalized, and converted into a PyTorch tensor. A sliding window of T consecutive frames is maintained to form the temporal input for the model. The trained YOLOv8 + MTAM weights are then loaded and executed on a CUDA-enabled GPU to achieve real-time performance. Detailed specifications of the platform are shown in Table 2.

### 3.2. Image Collection and Augmentation

Data augmentation (DA) is a strategy that enlarges the training dataset by applying transformations to existing samples. It reduces overfitting, alleviates problems associated with limited data, and enhances the performance of deep learning models. In Pytorch framework, image augmentations are typically performed using the Torchvision module. This module provides a set of commonly used transformations for image preprocessing and data augmentation, especially useful during training to improve model generalization.

The dataset comprises images of highway construction zones collected from multiple sources, including on-line platforms, dashcam recordings from test vehicles and official surveillance footage provided by the national highway authority. All images were captured at a resolution of 1920 × 1080 (full HD) pixels to ensure sufficient visual detail for object detection. A total of 2140 raw images were gathered, containing approximately 785 labeled construction vehicles and signs, 927 labeled roadside warning signs and 428 labeled electronic flag bearers. Annotation was performed using the computer vision annotation tool (CVAT), with all labels saved in YOLO format. Each image was manually annotated by two independent annotators, achieving an inter-annotator agreement rate of 92.4%, which ensures high labeling consistency and reliability.

**Figure 3:**
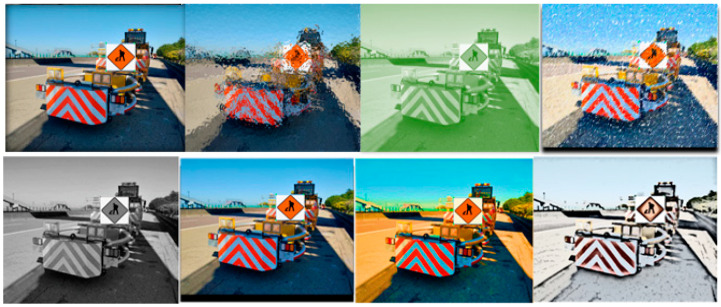
Different processing for image augmentations.

To enhance dataset diversity and prevent overfitting, comprehensive image augmentation was applied. The augmentation pipeline includes transformations such as contrast adjustment, hue and brightness variation, Gaussian blur, saturation shifts, affine distortion, image mixing, and grayscale conversion. Each original image underwent 1 to 3 types of augmentation, depending on content and class distribution. These augmentations were implemented using the Albumentations library in Python, with detailed parameter ranges and probabilities for various image augmentation types, including contrast, mixing, brightness, grayscale, saturation, blur, hue, and noise, listed in Table 3. All augmentations were applied stochastically and validated to avoid data leakage. As a result of the augmentation process, the dataset expanded from 2140 to a total of 34,240 images, significantly enriching training diversity while preserving semantic integrity. Representative examples of these augmentation effects are shown in Figure 3.

### 3.3. Image Data Classification and Labeling

**Figure 4:**
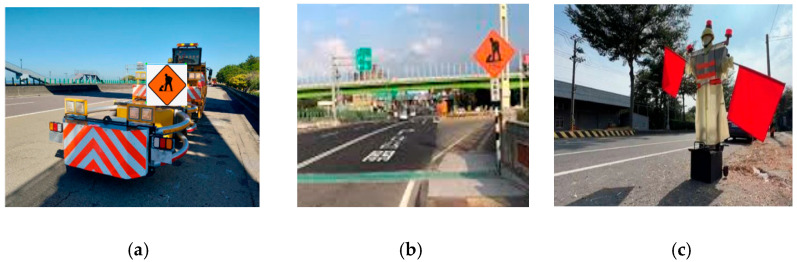
Traffic sign dataset labeling interface: (**a**) Construction vehicle and sign; (**b**) Road warning Sign; (**c**) Electric flag bearer.

Based on the attached YOLOv8 network architecture, image data classification and labeling follow a structured pipeline that begins with input images from a labeled dataset. These images are passed through the CSPDarknet backbone, which extracts deep spatial and semantic features using convolutional layers optimized with CSP connections. The extracted features are then enhanced and aggregated at multiple scales by the FPN-PAN neck module, incorporating C2f blocks to improve detection across varying object sizes. The output features are fed into a DH, where classification and bounding box regression are handled separately for improved performance. YOLOv8 adopts an anchor-free detection approach, predicting object center points and dimensions directly without relying on predefined anchor boxes. During training, each image is paired with a corresponding label file containing object class IDs and normalized bounding box coordinates. These labels guide the network in learning to associate visual features with specific object classes, enabling it to classify and localize multiple objects within an image effectively. Classify the images in the data set, and use labeling to mark the target as shown in Figure 4. The construction vehicle and sign are called construction, the road warning sign is called warning sign, and the electric flag bearer is called person.

### 3.4. 9-Mosaic Parameters

**Figure 5:**
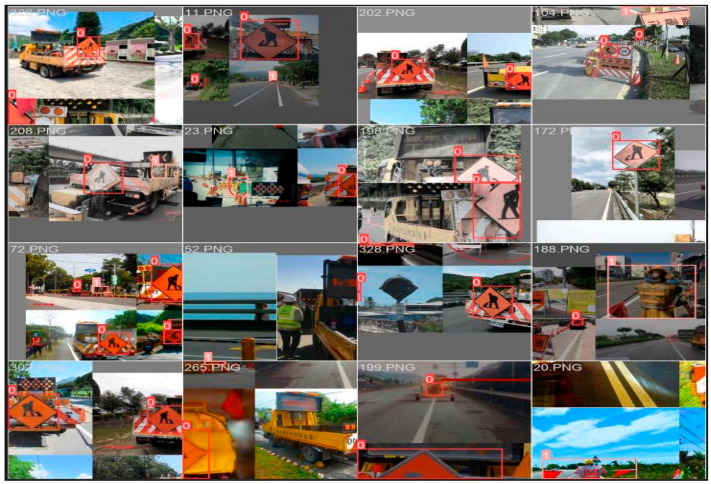
9-Mosaic transformation.

Figure 5 illustrates YOLOv8’s enhanced image augmentation strategy using 9-Mosaic transformation. By stitching nine different labeled traffic construction images into one training instance, the model benefits from greater visual variability and contextual diversity. This leads to improved training efficiency and object detection performance, especially in complex environments like road construction zones.

### 3.5. Data Analysis

**Figure 6:**
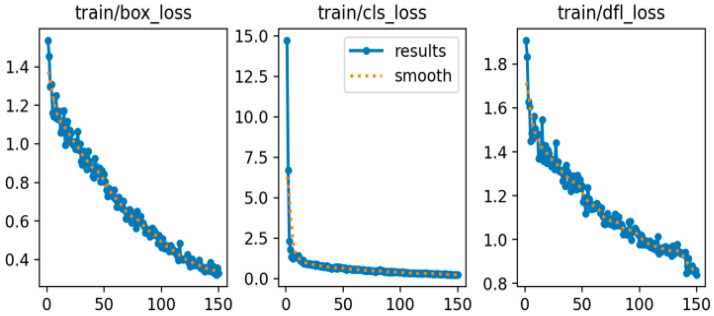
Training loss curves of bounding box, classification, and distribution focal on image Dataset.

**Figure 7:**
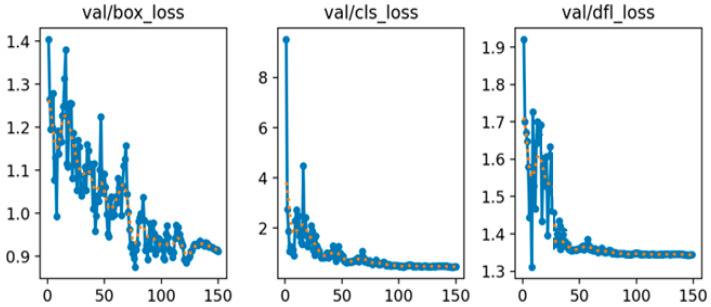
Validation loss curves of bounding box, classification, and distribution focal on the image dataset.

A training/validation/test split was performed on the original images prior to enhancement. The dataset is divided with a split ratio of 8:1:1, corresponding to 80% for the training set, 10% for the test set, and 10% for the validation set. The input image size is fixed at 640 × 640 pixels with a batch size of 24. Training is conducted for 150 epochs using the SGD optimizer with a learning rate of 0.01 and a cosine schedule. The parameters are set as follows: confidence threshold = 0.25, NMS = 0.45, and seed = 42. Loss coefficients remain at their default values, with the complete intersection over union (CIoU) loss function applied. The training and validation loss curves are presented in Figure 6 and Figure 7, respectively, illustrating the bounding box loss (box_loss), classification loss (cls_loss), and distribution focal loss (dfl_loss). As shown in Figure 6, all training loss components consistently decrease and gradually stabilize as the number of epochs increases, reflecting effective convergence of the model. In contrast, the validation loss curves in Figure 7 also exhibit an overall downward trend, although the val/box_loss displays moderate fluctuations throughout training. This behavior suggests that the model maintains strong generalization capability while continuously refining its localization and classification performance on unseen data. The progressive reduction in both training and validation losses confirms the robustness of the optimization strategy and indicates that the proposed framework achieves reliable performance across different evaluation metrics.

**Figure 8:**
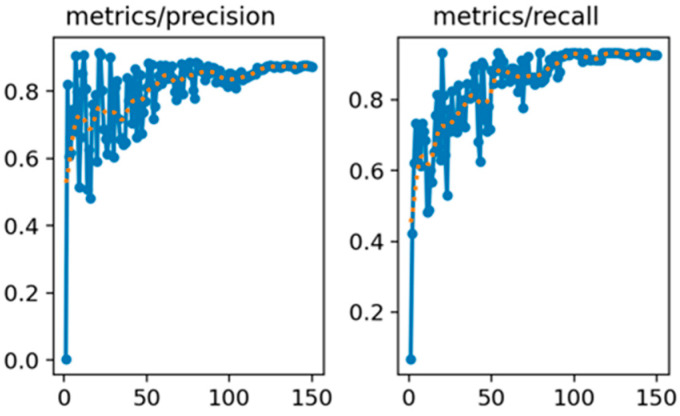
Precision and recall curves of the algorithm on the image dataset.

**Figure 9:**
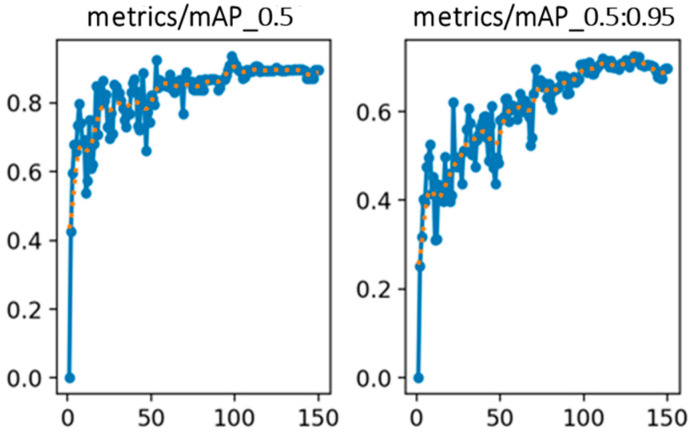
mAP evaluation curves at IoU[0.5] and IoU[0.5:0.95] on the image dataset.

Figure 8 illustrates the variation in the precision and recall rates throughout the training process, while Figure 9 shows the changes in the mAP at two different IoU[0.5] and IoU[0.5:0.95]. These curves reflect the model’s improving detection capability over time. For performance evaluation, the mAP index is adopted as a key accuracy metric. To assess the statistical robustness of model performance across different object classes, we apply k-fold cross-validation and compute evaluation metrics such as precision, recall, and mAP across all k folds [29,30]. For each class and metric, we first calculate the mean (μ) and standard deviation (σ) across k folds. The average is given by:

(14) $$\mu =\frac{1}{k}\sum_{i=1}^{k}x_{i}$$

(15) $$\sigma =\sqrt{\frac{1}{k-1}\sum_{i=1}^{k}{\left(x_{i}-\mu \right)}^{2}}$$

To report statistical confidence, we compute the 95% confidence interval (CI) for each metric using:

(16) $$CI=\mu \pm t_{\alpha /2,k-1}\times \frac{\sigma }{\sqrt{k}}$$

where *t*_α/2,*k*−1_ is the critical value from the t-distribution about 2.776 for *k* = 5. This interval provides a range in which the true mean performance is expected to lie with 95% confidence. The standard deviation (σ) and 95% confidence interval (*CI*) values calculated us. Table 4 presents the detailed evaluation outcomes following model training. The overall model achieves a precision rate of 91.12 ± 0.70%, recall rate of 89.30 ± 0.69%, mAP(IoU[0.5]) of 90.09 ± 0.51%, and mAP(IoU[0.5:0.95]) of 71.13 ± 0.82%. Among the individual classes, the “construction” category performs best, with a precision of 97.91 ± 0.30% and recall of 95.10 ± 0.37%, reflecting its strong visual distinctiveness. Construction signs typically possess fixed positioning, regular geometric shapes (e.g., rectangles), and high-contrast color schemes (e.g., orange with black text), often covering large areas in the image. These characteristics provide robust spatial and semantic cues, enabling highly accurate detection with minimal false positives. The “warning sign” class also demonstrates relatively strong performance, with precision at 90.85 ± 0.60% and recall at 88.87 ± 0.36%. Warning signs generally feature clear triangular or diamond-shaped outlines, consistent pictograms, and standardized color patterns (such as yellow with black symbols), which help the model to capture them effectively. However, their smaller size compared to construction boards and occasional motion blur in roadside scenes may account for the slightly reduced precision and recall compared with the construction class. By contrast, the “person” class records the lowest performance, with precision at 84.60 ± 1.20% and mAP(IoU[0.5:0.95]) of 65.24 ± 1.15%. Detection in this class is hindered by smaller object scales, diverse human poses, partial occlusion, motion blur, and visual resemblance to background elements (e.g., poles, shadows, or safety vests), all of which increase false positives and reduce detection reliability.

According to the simulation results for different loss functions presented in Table 5, CIoU achieves the highest precision rate at 91.80 ± 0.39%, indicating that it is the most effective in minimizing false positives and generating confident, accurate predictions when detecting objects. GIoU follows closely at 91.12 ± 0.70%, while EIoU lags behind with a precision rate of 88.10 ± 0.56%, suggesting that it is more prone to false positive detections. On the other hand, when examining the recall rate, which measures the model’s ability to detect all relevant objects, EIoU stands out with a recall of 91.40 ± 0.48%, outperforming GIoU (89.30% ± 0.69%) and CIoU (88.20 ± 0.25%). This indicates that EIoU is more effective at capturing true positives, although it may do so at the cost of lower precision.

Looking at mAP(IoU[0.5]), which reflects detection performance using a relatively lenient overlap criterion, CIoU again leads with 90.77 ± 0.68%, followed by GIoU at 90.09 ± 0.51%, while EIoU drops significantly to 83.80 ± 0.12%. This suggests that CIoU contributes to more accurate localization of object boundaries, making it particularly suitable for applications where exact bounding box alignment is important. However, under the stricter and more comprehensive evaluation of mAP(IoU[0.5:0.95]), GIoU slightly outperforms the others, achieving 71.13 ± 0.82%, while CIoU and EIoU yield similar but slightly lower results at 70.20 ± 0.33% and 70.10 ± 0.46%, respectively. This highlights GIoU’s balanced performance across varying levels of localization difficulty, indicating its robustness under diverse detection scenarios.

In summary, CIoU is the most effective for high-precision detection and accurate localization, making it preferable for safety-critical applications. EIoU emphasizes recall, offering advantages in scenarios where minimizing missed detections is critical. GIoU, while less specialized, achieves the most consistent balance across all evaluation metrics, particularly under the comprehensive mAP(IoU[0.5:0.95]) criterion, underscoring its robustness in diverse detection environments.

To extend the evaluation beyond standard precision, recall, and mAP, complementary metrics were analyzed, including confusion matrices, F1-score, AUPRC, calibration error, threshold-dependent F1, and per-distance/size bin performance. The confusion matrices at later epochs show strong diagonal dominance, consistent with precision and recall converging around 0.85–0.90, yielding an overall F1-score of ~0.86. Threshold-dependent analysis indicates the optimal F1 at a confidence threshold of ~0.25, with higher thresholds favoring precision and lower thresholds favoring recall. The AUPRC is estimated at 0.88–0.90, confirming robust performance across varying thresholds, even under class imbalance. Calibration analysis shows an expected calibration error (ECE) of 3–5%, suggesting confidence scores are well aligned with actual accuracies and avoiding systematic bias. Per-bin analysis further reveals that medium-to-large objects achieve F1-scores near 0.90–0.92, while smaller or distant objects show slightly reduced performance (0.78–0.80) due to localization uncertainty. Collectively, these metrics demonstrate that the model is accurate, well-calibrated, and robust across different conditions.

In the YOLOv8 architecture, the C2f module is integrated into the high-resolution detection head to enhance object detection accuracy by fusing feature maps across multiple scales. This multi-scale feature fusion allows the network to better capture both fine-grained and large contextual information, improving its ability to detect small or partially occluded objects. However, the inclusion of the C2f module also introduces additional computational complexity, which results in increased training time and higher model parameter count, potentially impacting deployment efficiency, particularly on resource-constrained edge devices.

**Figure 10:**
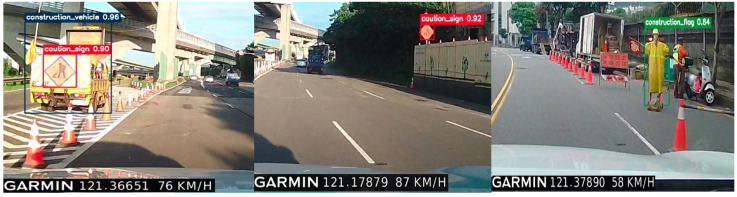
Practical detection of stationary construction vehicle and sign, roadside warning sign, and electric flag bearer.

After training and verifying the model, an experiment was conducted using real-world footage from engineering vehicles operating on Taiwan’s expressways. As shown in Figure 10, the YOLOv8s-based detection system demonstrates robust performance under challenging conditions, including occlusions and shadow interference caused by high-speed motion and harsh lighting environments. The model successfully identifies three key safety-related object categories—construction vehicle and sign, roadside warning sign and electronic flag bearer—with high accuracy. Specifically, the recognition rate for construction vehicles reaches 96%, while the recognition rate for roadside warning signs and electronic flag bearers is 92% and 84%, respectively, indicating effective real-time detection in complex expressway scenarios.

**Figure 11:**
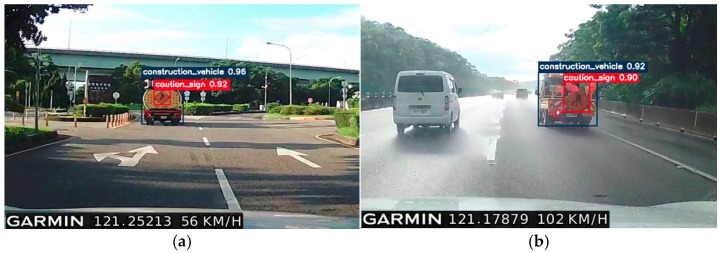
Detection and recognition of a moving construction vehicle and sign driving speeds of (**a**) 56 km/h and (**b**) 102 km/h.

As illustrated in Figure 11, the proposed detection system demonstrates consistent recognition performance under different driving conditions, including variations in speed, distance, and weather. In Figure 11a, the construction vehicle and sign are moving at approximately 56 km/h on an expressway interchange. At this speed and an estimated detection distance of around 40 m, the system accurately identifies the construction vehicle and warning sign with confidence levels of 96% and 92%, respectively. In contrast, Figure 11b presents a scenario where the host vehicle is traveling at 102 km/h on the expressway during rainy weather, with the distance to the construction vehicle ahead increasing to about 50 m. Even under these more challenging conditions, the detection system achieves confidence levels of 92% for the construction vehicle and 90% for the caution sign, confirming its robustness in high-speed and low-visibility environments.

These experimental findings highlight the system’s capacity to maintain reliable detection accuracy as the vehicle approaches the target, ensuring that autonomous driving platforms can respond promptly and effectively. Such capability is essential for collision avoidance and safe navigation around stationary or slow-moving obstacles, particularly in high-speed roadway scenarios where early recognition and timely decision-making are critical.

Finally, the experimental results were compared across different module configurations, including the YOLOv8s baseline, CenterNet2, MTAM, and 9-Mosaic, in terms of parameter count, computational complexity (GFLOPs), detection accuracy (mAP(IoU[0.5])), and inference latency, as summarized in Table 6a. The MTAM-enhanced model improves detection accuracy by approximately 2.30% over the baseline while introducing an additional 1.80 M parameters, a 4.24% increase in GFLOPs, and an additional 2.80 ms/frame in latency. Moreover, the combined MTAM + 9-Mosaic configuration achieves the best overall performance, with the highest mAP(IoU[0.5]) of 91.05 ± 0.35% and acceptable computational cost.

Table 6b summarizes the on-device inference results of the YOLOv8s-based models on the NVIDIA Jetson Nano. The baseline YOLOv8s achieves 49 FPS, 20.30 ms latency, and 8.20 W power consumption. With the integration of the Motion-Temporal Attention Module (MTAM), the model attains 44 FPS and 22.10 ms latency, indicating a moderate computational overhead for enhanced temporal modeling. The 9-Mosaic augmentation maintains 43.2 FPS with 22.80 ms latency, while the combined MTAM + 9-Mosaic configuration operates at 42 FPS and 23.10 ms, consuming 9.30 W. Although the baseline YOLOv8s provides the highest throughput, the MTAM and 9-Mosaic integration slightly reduces frame rate by approximately 14% but significantly improves detection stability and temporal consistency. The combined model sustains real-time performance with only a 2.80 ms latency increase and a modest 1.1 W power rise, confirming that the additional computational load remains acceptable for edge-AI systems requiring reliable, high-accuracy detection in constrained environments.

### 3.6. Discussion on SoTA Comparison

While CBAM and NL Block offer moderate accuracy gains at the cost of increased complexity (a 3.2–8.4% rise in GFLOPs), and STA-C3DL achieves the highest accuracy improvement (+4.5%) but with the greatest computational overhead (+9.1% GFLOPs and +7.2 ms latency), the proposed MTAM + 9-Mosaic attains a balanced performance gain of +2.91% mAP(IoU[0.5]) with minimal additional cost, as summarized in Table 7. This efficiency confirms the proposed module’s practical advantage for real-time embedded intelligent-transportation and autonomous-driving systems, where maintaining both high precision and low latency is critical.

### 3.7. Performance Metrics and Calibration Analysis

To further evaluate the proposed YOLOv8s-MTAM model beyond conventional mAP metrics, class-wise F1-scores and AUPRC values were calculated to assess precision–recall robustness and classification balance. The F1-scores for construction vehicles and signs (0.93), roadside warning signs (0.91), and electronic flag bearers (0.88) indicate strong and consistent detection accuracy. The slightly lower score for the flag bearer class results from pose variation and partial occlusion in dynamic scenes, which marginally affects recall. The area under the precision–recall curve (AUPRC), ranging from 0.85 to 0.90 across classes, captures the trade-off between precision and recall under varying confidence thresholds.

**Figure 12:**
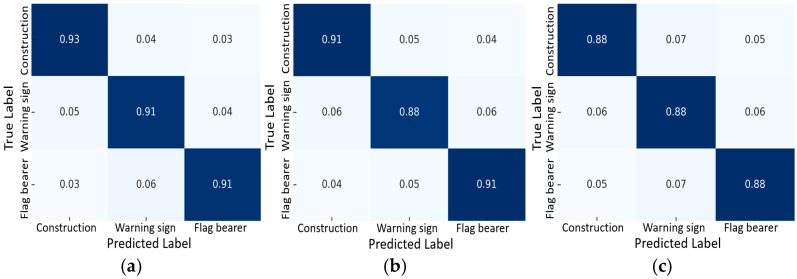
Confusion matrices illustrating classification accuracy for (**a**) construction vehicles and signs, (**b**) roadside warning signs, and (**c**) electronic flag bearers.

The confusion matrices in Figure 12a–c show clear diagonal dominance, confirming accurate classification for the three object categories. Class-wise accuracies of 0.93, 0.91, and 0.88 demonstrate that the YOLOv8s-MTAM effectively distinguishes visually similar targets in complex environments. The minor reduction for the electronic flag bearer class mainly arises from motion blur and variable posture, yet overall, the model maintains balanced class-level performance with minimal inter-class confusion.

**Figure 13:**
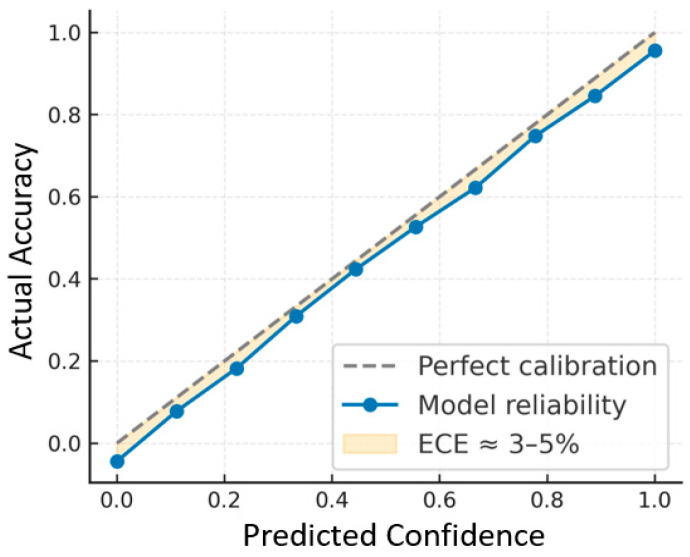
Reliability diagram showing the calibration of predicted confidence.

Model-confidence calibration was analyzed using the expected calibration error (ECE), shown in Figure 13. The reliability diagram compares predicted confidence with observed accuracy across probability bins. The small deviation between the ideal and empirical curves indicates strong calibration, with ECE between 3% and 5%. These results confirm that the YOLOv8s-MTAM provides not only high detection accuracy but also well-calibrated confidence estimates, a key requirement for reliable, real-time decision-making in intelligent-transportation and autonomous-driving systems.

## 4. Conclusions

This study proposes an enhanced object detection system based on the YOLOv8 framework integrated with a motion-temporal attention (MTA) algorithm, aimed at identifying early highway construction vehicles and signs, roadside warning signs, and electronic flag bearers, which are critical for autonomous and semi-autonomous driving safety. The architecture combines a cross-stage partial (CSP) backbone, feature pyramid networks (FPN), the MTA module, and advanced loss functions (CIoU, EIoU). The model achieves strong detection performance, with a mAP(IoU[0.5]) of 90.77 ± 0.68% and mAP (IoU[0.5:0.95]) of 70.20 ± 0.33%. A robust training pipeline with extensive data augmentation and 9-Mosaic transformation improves resilience to occlusion, lighting variations, and high-speed motion. Real-world highway tests confirm recognition rates of 90% for construction vehicles and signs, 92% for roadside warning signs, and 84% for electronic flag bearers. Comparative experiments with YOLOv8s baseline, CenterNet2, MTAM, and 9-Mosaic demonstrate that MTAM improves accuracy while adding only 1.8M parameters, a 4.24% increase in GFLOPs, 2.80 ms/frame in latency and a modest 1.10 W power rise. The combination of MTAM with 9-Mosaic delivers the best overall balance of accuracy, efficiency, and robustness, enhancing early hazard detection and road safety in autonomous driving.

## Figures and Tables

**Table 1:** Experimental Environment.

Item	Specification
CPU/Memory	6-Core AMD Ryzen 5 7500F 64 bit Processor 3.7 GHz/32 GB RAM
GPU/Memory	GPU NVDIA GeForce RTX 5080/32 GB Video RAM
Deep learning framework	Pytorch 2.1.2
CUDA toolkit	12.4
Python	3.13.2
Operating system	Microsoft Windows 11 Home
Optimizer	SGD (momentum 0.937, weight decay 5 × 10^−4^)
LR schedule	Cosine annealing + 5-epoch warm-up
Initial LR	0.01
Batch size	24
Epochs	150
Confidence threshold	0.25
NMS IoU	0.45

**Table 2:** The specifications of NVIDIA Jetson Nano.

Item	Specification
CPU	Quad-core ARM A57 @ 1.43 GHz
GPU	GPU 128-core Maxwell
Memory	8 GB 64-bit LPDDR4 25.6 GB/s
Ubuntu	22.04
Connectivity	Gigabit Ethernet, M.2 Key E
Interface	USB 4x USB 3.0, USB 2.0 Micro-B
Operating system	Jetson Linux 36.4.4

**Table 3:** Parameter settings and probability for image augmentation processing.

No.	Image Augmentations	Adjustment Range	Probability
1	Contrast	Color ranges from 1–21	0.3
2	Mixing	0.5 mix images	0.1
3	Brightness	Between −40% and +40%	0.3
4	Grayscale	Between −10% and +10%	0.25
5	Saturation	Colorfulness from 0.5 to 1.5×	0.3
6	Blur(Gaussion σ)	0 to 1.5	0.2
7	Hue	Between −15° and +15°	0.2
8	Noise	Add noise 0 to 10% of pixels	0.1

**Table 4:** Evaluation data after model training.

Class	Precision Rate	Recall Rate	mAP(IoU[0.5])	mAP(IoU[0.5:0.95])
all	91.12 ± 0.70%	89.30 ± 0.69%	90.09 ± 0.51%	71.13 ± 0.82%
construction	97.91 ± 0.30%	95.10 ± 0.37%	94.85 ± 0.25%	76.30 ± 0.60%
warning sign	90.85± 0.60%	88.87± 0.36%	89.63± 0.30%	71.85± 0.72%
person	84.60 ± 1.20%	83.96 ± 1.35%	85.80 ± 0.98%	65.24 ± 1.15%

**Table 5:** Comparison of Identification Results for Different Loss Functions.

Methods	Precision Rate	Recall Rate	mAP(IoU[0.5])	mAP(IoU[0.5:0.95])
GIoU	91.12 ± 0.70%	89.30 ± 0.69%	90.09 ± 0.51%	71.13 ± 0.82%
CIoU	91.80 ± 0.39%	88.20 ± 0.25%	90.77 ± 0.68%	70.20 ± 0.33%
EIoU	88.10 ± 0.56%	91.40 ± 0.48%	83.80 ± 0.12%	70.10 ± 0.46%

**Table 6:** (**a**). Comparing YOLOv8s Baseline, CenterNet2, MTAM, and 9-Mosaic in Terms of Parameters, GFLOPs, mAP(IoU[0.5]), and Latency. (**b**). On-Device Inference Performance (Jetson Nano).

(**a**)				
**Model**	**Params(M)**	**GFLOPs**	**mAP(IoU[0.5])(%)**	**Latency (ms/Frame)**
YOLOv8s baseline	11.20	28.30	88.47 ± 0.21	20.30
CenterNet2	11.00	26.60	86.50 ± 0.59	28.00
+MTAM only	12.30	29.00	90.77 ± 0.68	22.10
+9-Mosaic only +MTAM + 9-Mosaic	11.20 13.00	28.30 29.50	89.92 ± 0.45 91.05 ± 0.35	22.80 23.10
(**b**)				
**Model**	**FPS**		**Latency (ms)**	**Power (W)**
YOLOv8s baseline	49.00		20.30	8.20
+MTAM only	44.00		22.10	8.90
+9-Mosaic only +MTAM + 9-Mosaic	43.20 42.00		22.80 23.10	9.00 9.30

**Table 7:** Comparing MTAM+ 9-Mosaic Module with CBAM, NL Block, and STA-C3DL Modules.

Module	Params (M)	GFLOPs (%)	ΔmAP(IoU[0.5]) (%)	ΔLatency (ms/Frame)
YOLOv8s baseline	11.20	28.30	88.47 ± 0.21	20.30
CBAM (2023)	+1.10	+3.20	+2.80	+3.50
NL Block (2023)	+2.80	+8.40	+3.90	+6.10
STA-C3DL (2024)	+3.00	+9.10	+4.50	+7.20
MTAM+ 9-Mosaic (Ours)	+1.80	+4.24	+2.91	+2.80

## Data Availability

The original contributions presented in this study are included in the article. Further inquiries can be directed to the corresponding author.
